# Mitochondrial DNA evidence reflects high genetic divergence of *Amynthas aspergillum* (Oligochaeta: Megascolecidae) in southern China

**DOI:** 10.1002/ece3.11452

**Published:** 2024-05-31

**Authors:** Jiali Li, Jibao Jiang, Qing Jin, Zhu Yuan, Jiangping Qiu

**Affiliations:** ^1^ School of Agriculture and Biology Shanghai Jiao Tong University Shanghai China; ^2^ School of Pharmacy Shanghai University of Medicine & Health Sciences Shanghai China

**Keywords:** *Amynthas aspergillum*, earthworms, genetic differentiation, mitochondrial DNA, population genetic structure

## Abstract

*Amynthas aspergillum* (Perrier, 1872), a natural resource used in traditional Chinese medicine (Guang‐dilong) with high economic value, is widely distributed in forests and farmland habitats in the hilly areas of southern China. To investigate the extent of genetic differentiation and diversity in *A. aspergillum*, a population genetic structure study was performed on 157 samples from 75 locations in southern China using the mitochondrial genes *COI*, *COII*, *12S rRNA*, *16S rRNA*, and *NDI*. The results indicated that *A. aspergillum* had a high level of genetic diversity, and variation within populations was the main source of the total variation. Six deeply divergent mitochondrial clades (I–VI) were detected using both phylogenetic tree and haplotype network analyses. This finding was supported by the high Kimura two‐parameter genetic distance and the pairwise fixation index value obtained based on the *COI* gene. No significant phylogeographic structures were observed. The widespread geographic distribution of clades II, IV, and VI suggested a recent demographic expansion based on multiple analysis results. These results include a high level of Hd and low *π*, star‐shaped haplotype network structures with a high number of less frequent haplotypes, significantly negative neutrality test values, and a unimodal mismatch distribution pattern. The divergence time estimates and reconstruction of the ancestral area revealed that *A. aspergillum* originated in Guangxi Province and underwent initial intraspecific diversification in the early Pliocene to generate clade I. Then, it gradually dispersed eastward and rapidly differentiated into clades II–V during the Pleistocene. The Yunnan‐Guizhou Plateau and Nanling and Wuyi Mountains might act as geographical barriers for the spread of *A. aspergillum* to the west and north.

## 1 INTRODUCTION

Earthworms, the largest biomass among the invertebrates living in soil, are considered as soil ecosystem engineers (Jones et al., 1996). Earthworms play a significant role in soil ecosystems by mixing soils, improving soil structure and fertility, and enhancing the soil nutrient cycle and biological growth (Blouin et al., 2013; Brown et al., 2004). Similar to many soil invertebrates, earthworms exhibit weak active diffusion and are often distributed globally, leading to substantial genetic polymorphisms. Therefore, the genetic differentiation and diffusion of earthworms are susceptible to geological movements, environmental conditions, and human activities. This susceptibility makes earthworms an ideal model for phylogeographical studies (Fernández et al., 2013, 2011; Seesamut et al., 2019; Yuan et al., 2020; Zhao et al., 2015).

Molecular, phylogenetic, and population genetic studies can shed light on past biogeographic events and unveil life history features that contribute to shaping the distribution of genetic variation among populations (Searle, 2000). Molecular genetic data, including those obtained from mitochondrial genes (Aspe & James, 2018), nuclear genes (Novo et al., 2014), and microsatellites (Somers et al., 2011), have been used to explore the evolutionary history of species and populations through phylogenetic, phylogeographic, and population‐level approaches. These investigations have identified changes in the ecological environment as the primary factors affecting earthworm genetic diversity. Mitochondrial DNA (mtDNA) has become one of the most common molecular markers in the study of earthworm genetic evolution. This is because of its unique advantages, such as simple genetic structure, lack of genetic recombination, and maternal inheritance (Brown et al., 1979; Harrison, 1989). Chang and James summarized previous studies and observed many selected gene sequences were either too short or evolved too slowly. The findings suggest that long sequences (>2000 bp) created by combining genes with different evolutionary rates are crucial for analyses at different taxonomic levels (Chang & James, 2011). Recent studies have demonstrated that combining genetic data produces a more objective phylogenetic tree than using a single gene for exploring phylogenetic relationships among species or populations (Dong et al., 2020; Fernández et al., 2016; Klein et al., 2020; Sun et al., 2017).

**FIGURE 1:**
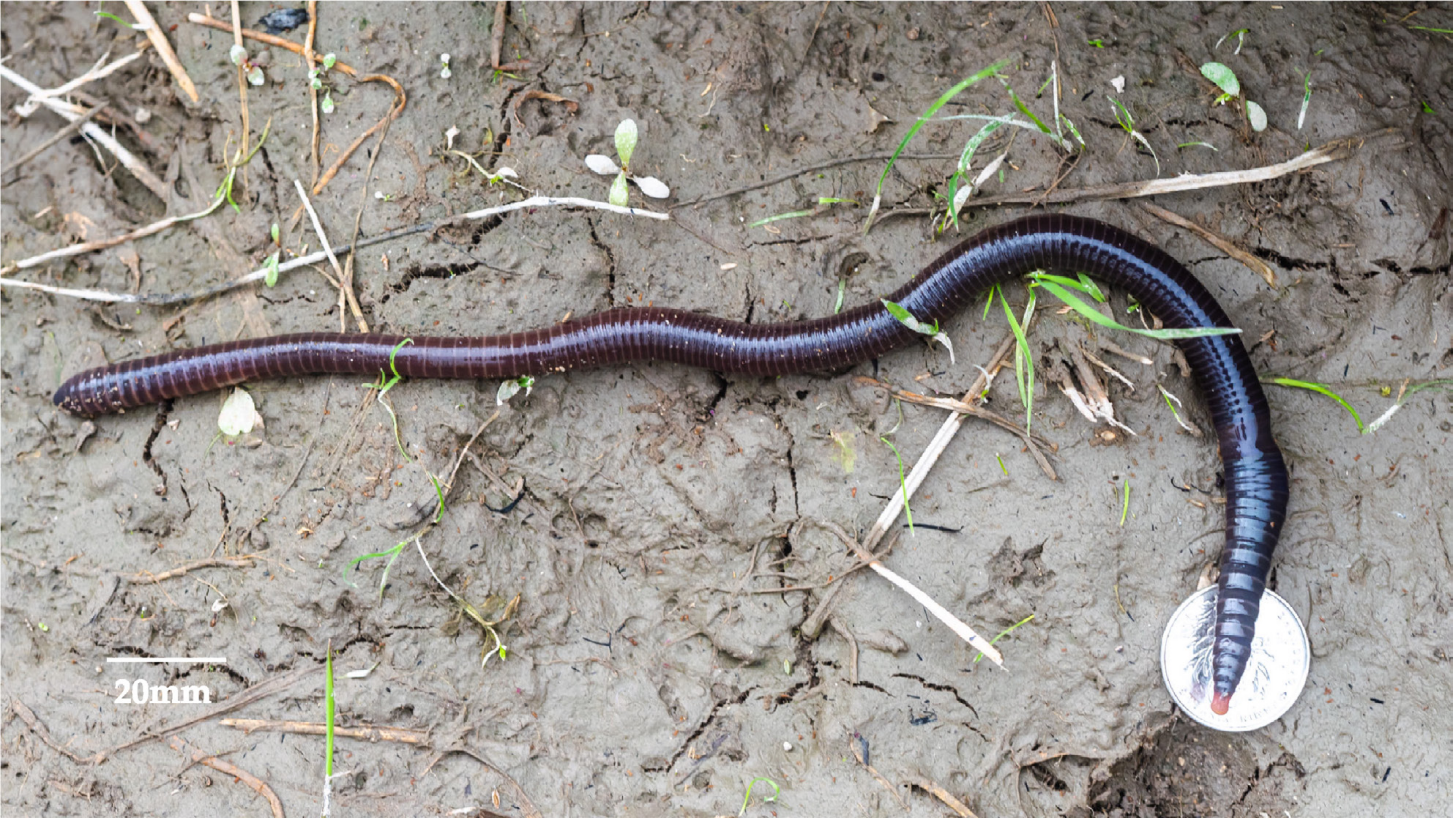
Adult *Amynthas aspergillum* found in the field in Fujian Province. The photo was taken by Jibao Jiang.

The model earthworm species, *Amynthas aspergillum* (Perrier, 1872) (Figure 1), belongs to the genus *Amynthas* of the family Megascolecidae. This species is abundant in forest and farmland habitats, mainly distributed in southern China and Vietnam (Jiang & Qiu, 2018). In China, *A. aspergillum* is an important source of traditional Chinese medicine in the form of Guang‐dilong, the dried body of *A. aspergillum*. Additionally, it is one of four medicinal earthworm species explicitly included in the Chinese Pharmacopeia (Huang et al., 2016, 2019; Sun et al., 2019). Although few studies have examined the phylogeny and genetic structure of *A. aspergillum*, their exact genetic relationships have not been determined because of limited sampling (Jiang & Qiu, 2018).

Therefore, the present study aimed to reveal the genetic divergence and structure of *A. aspergillum* populations sampled from southern China. *A. aspergillum* is mostly distributed in the hilly and mountainous areas of southern China, and has a long history of use in China as a traditional Chinese medicine resource with high economic value. This leads to the hypothesis that *A. aspergillum* has high genetic diversity and differentiation, but no obvious phylogeographic structures. To test this hypothesis, the molecular approach based on mitochondrial genes cytochrome c oxidase subunit I (COI), cytochrome oxidase subunit II (COII), 12S ribosomal (r)RNA (12S rRNA), 16S rRNA (16S rRNA), and NADH dehydrogenase subunit I (NDI) was used to determine: (1) the genetic variability and divergence of *A. aspergillum*; (2) the population structure and phylogeography of *A. aspergillum*; and (3) the origin, dispersal and demographic history of *A. aspergillum*.

## 2 MATERIALS AND METHODS

### 2.1 Sample collection and DNA extraction

**FIGURE 2:**
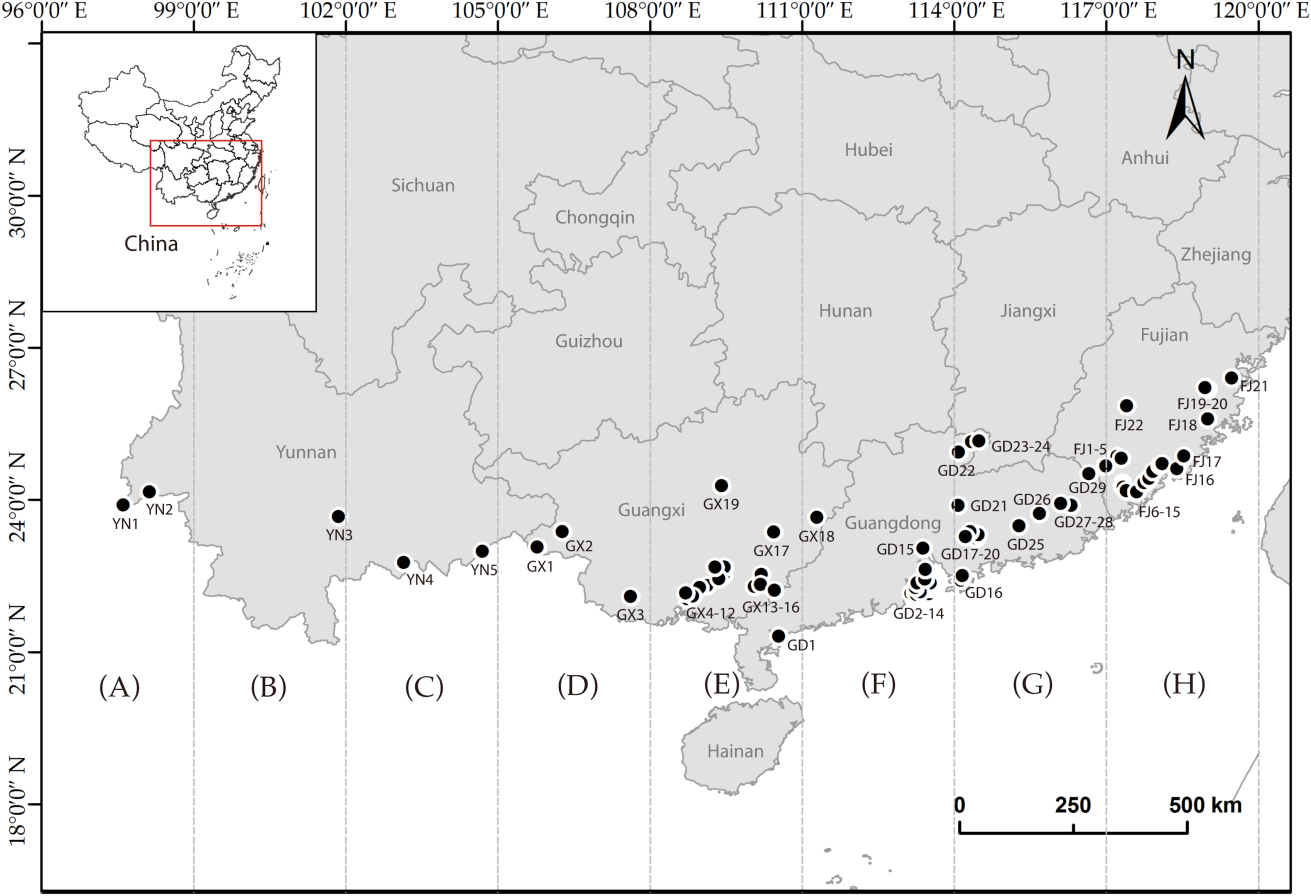
Map of the sampled populations of *Amynthas aspergillum* in southern China. Location details are provided in Table 1.

In total, 157 *A. aspergillum* individuals were collected from 75 locations in southern China (Figure 2 and Table 1). In the field, the collected sample earthworms were anesthetized by immersion in 10% ethyl‐alcohol solution and then fixed and preserved in 95% ethanol solution for molecular analysis. Muscle fragments (approximately 2 mm) were dissected from the tails of the specimens for DNA extraction. Total genomic DNA was extracted using the E.Z.N.A.® Mollusk DNA kit (Omega Bio‐Tek, Norcross, GA, USA) following the manufacturer's protocol for invertebrate tissue samples.

**TABLE 1:** Sampling localities of *Amynthas aspergillum* and their GPS coordinates.

No.	Location	Abb.	Region	Date	Number	GPS coordinates	Altitude (m)
1	Ruili City, Yunnan province	YN1	A	2017	1	97.6075° E, 23.8970° N	752
2	Mang City, Yunnan province	YN2	A	2021	3	98.1254° E, 24.1580° N	857
3	Yuanjiang City, Yunnan province	YN3	B	2011	1	101.8514° E, 23.6692° N	854
4	Jinping Miao, Yao, and Dai Autonomous County, Yunnan province	YN4	C	2021	4	103.1443° E, 22.7636° N	504
5	Malipo County, Yunnan province	YN5	C	2021	3	104.6956° E, 22.9833° N	1104
6	Napo County, Guangxi province	GX1	D	2021	2	105.7764° E, 23.0692° N	277
7	Jingxi City, Guangxi province	GX2	D	2021	2	106.2693° E, 23.3763° N	817
8	Ningming County, Guangxi province	GX3	D	2021	3	107.6182° E, 22.0932° N	145
9	Qinzhou City, Guangxi province	GX4	E	2021	1	108.7058° E, 22.1622° N	58
10	Qinzhou City, Guangxi province	GX5	E	2021	5	108.7352° E, 22.0503° N	8
11	Qinzhou City, Guangxi province	GX6	E	2021	3	108.8401° E, 22.1025° N	31
12	Lingshan County, Guangxi province	GX7	E	2021	2	108.9754° E, 22.2690° N	35
13	Lingshan County, Guangxi province	GX8	E	2021	1	109.1219° E, 22.3042° N	52
14	Lingshan County, Guangxi province	GX9	E	2021	4	109.3557° E, 22.4433° N	69
15	Lingshan County, Guangxi province	GX10	E	2021	3	109.4529° E, 22.5157° N	112
16	Hengzhou City, Guangxi province	GX11	E	2021	9	109.2852° E, 22.6714° N	53
17	Hengzhou City, Guangxi province	GX12	E	2021	1	109.4662° E, 22.6691° N	62
18	Bobai County, Guangxi province	GX13	E	2021	3	110.0606° E, 22.2910° N	85
19	Luchuan County, Guangxi province	GX14	E	2021	1	110.1827° E, 22.3309° N	188
20	Luchuan County, Guangxi province	GX15	E	2021	2	110.1949° E, 22.5290° N	87
21	Beiliu City, Guangxi province	GX16	E	2021	2	110.4579° E, 22.2163° N	101
22	Pingnan County, Guangxi province	GX17	E	2021	3	110.4376° E, 23.3613° N	50
23	Wuzhou City, Guangxi province	GX18	F	2021	2	111.2912° E, 23.6500° N	37
24	Liuzhou City, Guangxi province	GX19	E	2010	1	109.4090° E, 24.2750° N	98
25	Wuchuan City, Guangdong province	GD1	E	2019	5	110.5379° E, 21.3096° N	3
26	Zhuhai City, Guangdong province	GD2	F	2022	1	113.1648° E, 22.0460° N	0
27	Zhuhai City, Guangdong province	GD3	F	2022	1	113.2137° E, 22.0868° N	1
28	Zhuhai City, Guangdong province	GD4	F	2022	1	113.1658° E, 22.1003° N	−7
29	Zhuhai City, Guangdong province	GD5	F	2022	3	113.4428° E, 22.1996° N	2
30	Zhuhai City, Guangdong province	GD6	F	2022	1	113.4856° E, 22.2183° N	8
31	Zhuhai City, Guangdong province	GD7	F	2022	1	113.2063° E, 22.2858° N	5
32	Zhuhai City, Guangdong province	GD8	F	2022	3	113.1952° E, 22.2559° N	11
33	Zhuhai City, Guangdong province	GD9	F	2022	4	113.1393° E, 22.1733° N	5
34	Zhuhai City, Guangdong province	GD10	F	2022	1	113.1883° E, 22.1510° N	21
35	Zhuhai City, Guangdong province	GD11	F	2022	1	113.2161° E, 22.1638° N	24
36	Zhuhai City, Guangdong province	GD12	F	2022	1	113.2402° E, 22.2781° N	7
37	Zhongshan City, Guangdong province	GD13	F	2022	1	113.3611° E, 22.3047° N	21
38	Zhongshan City, Guangdong province	GD14	F	2022	1	113.3509° E, 22.3781° N	33
39	Guangzhou City, Guangdong province	GD15	F	2011	1	113.3840° E, 23.0470° N	25
40	Tai Mo Shan, Hongkong	GD16	G	2016	5	114.1600° E, 22.5000° N	560
41	Boluo County, Guangdong province	GD17	G	2021	2	114.4355° E, 23.2431° N	43
42	Boluo County, Guangdong province	GD18	G	2021	2	114.4727° E, 23.3112° N	25
43	Boluo County, Guangdong province	GD19	G	2021	2	114.3163° E, 23.3688° N	67
44	Boluo County, Guangdong province	GD20	G	2021	1	114.2250° E, 23.2708° N	28
45	Longmen County, Guangdong province	GD21	G	2021	6	114.0740° E, 23.8894° N	210
46	Shixing County, Guangdong province	GD22	G	2021	3	114.0856° E, 24.9374° N	102
47	Nanxiong City, Guangdong province	GD23	G	2019	1	114.3439° E, 25.1454° N	142
48	Nanxiong City, Guangdong province	GD24	G	2019	1	114.4866° E, 25.1610° N	92
49	Heyuan City, Guangdong province	GD25	G	2011	1	115.2778° E, 23.4858° N	278
50	Wuhua County, Guangdong province	GD26	G	2021	1	115.6826° E, 23.7288° N	141
51	Fengshun County, Guangdong province	GD27	G	2021	1	116.3061° E, 23.8888° N	280
52	Fengshun County, Guangdong province	GD28	G	2021	1	116.1016° E, 23.9304° N	167
53	Dabu County, Guangdong province	GD29	G	2021	1	116.6551° E, 24.5106° N	67
54	Yongding Count, Fujian province	FJ1	H	2020	1	116.9725° E, 24.6648° N	390
55	Yongding Count, Fujian province	FJ2	H	2020	1	116.9915° E, 24.6675° N	416
56	Nanjing County, Fujian province	FJ3	H	2020	1	117.1727° E, 24.8337° N	455
57	Nanjing County, Fujian province	FJ4	H	2020	3	117.2141° E, 24.8578° N	244
58	Nanjing County, Fujian province	FJ5	H	2020	3	117.2951° E, 24.8165° N	128
59	Pinghe County, Fujian province.	FJ6	H	2020	2	117.3156° E, 24.3306° N	34
60	Pinghe County, Fujian province	FJ7	H	2020	3	117.3152° E, 24.2860° N	50
61	Pinghe County, Fujian province	FJ8	H	2020	5	117.3375° E, 24.2429° N	78
62	Pinghe County, Fujian province	FJ9	H	2020	2	117.3972° E, 24.1776° N	193
63	Zhangpu County, Fujian province	FJ10	H	2020	3	117.5974° E, 24.1548° N	35
64	Longhai City, Fujian province	FJ11	H	2020	1	117.7418° E, 24.3384° N	15
65	Longhai City, Fujian province	FJ12	H	2020	1	117.8419° E, 24.4220° N	25
66	Xiamen City, Fujian province	FJ13	H	2020	2	117.9174° E, 24.5606° N	25
67	Xiamen City, Fujian province	FJ14	H	2020	1	118.0597° E, 24.6789° N	41
68	Xiamen City, Fujian province	FJ15	H	2020	3	118.1016° E, 24.709° N	43
69	Nanan City, Fujian province	FJ16	H	2020	1	118.3961° E, 24.6154° N	20
70	Jinjiang City, Fujian province	FJ17	H	2020	1	118.5321° E, 24.8599° N	26
71	Putian City, Fujian province	FJ18	H	2020	1	119.0047° E, 25.5958° N	149
72	Minhou County, Fujian province	FJ19	H	2020	2	119.0025° E, 26.1868° N	24
73	Minhou County, Fujian province	FJ20	H	2020	2	118.9516° E, 26.2131° N	29
74	Lianjiang County, Fujian province	FJ21	H	2020	1	119.4760° E, 26.4018° N	115
75	Yongan City, Fujian province	FJ22	H	2020	1	117.4052° E, 25.8535° N	263

### 2.2 DNA amplification and sequencing

Polymerase chain reaction (PCR) was performed to amplify five fragments of the mitochondrial genome: *COI*, *COII*, *12S rRNA*, *16S rRNA*, and *NDI*. The primers used for PCR and sequencing used in this study are presented in Table 2. The PCR amplification mixture (50 μL) consisted of 1 μL DNA template, 2 μL of each primer, 35.4 μL double‐distilled H_2_O, and 9.6 μL TransTaq™ DNA Polymerase High Fidelity MixTaq containing 0.6 μL TransTaq™ HiFi DNA polymerase, 4 μL of 2.5 mM dNTPs, and 5 μL of 10× TransTaq™ HiFi Buffer I. The PCR cycling conditions were as follows: an initial activation step at 94°C for 5 min, followed by 32 amplification cycles of denaturation at 94°C for 30 s, annealing at 50°C for 30 s, elongation at 72°C for 60 s, and a final elongation step at 72°C for 10 min. The amplified PCR products were analyzed using 1% agarose gel electrophoresis, purified, and sequenced by the Beijing Genomics Institute, Shanghai Branch, China. The sequences have been submitted to GenBank under accession numbers *COI*: PP580956–PP581112; *COII*: PP588776–PP588932; *NDI*: PP593059–PP593215; *12S rRNA*: PP582003–PP582159; *16S rRNA*: PP581136–PP581292.

**TABLE 2:** Primers used for PCR and sequencing.

Gene	Primer	Sequence	Source
*COI*	LCO1490	GGTCAACAAATCATAAAGATATTGG	Folmer et al. (1994)
	HCO2198	TAAACTTCAGGGTGACCAAAAAATCA	Folmer et al. (1994)
	CO1‐E	TATACTTCTGGGTGTCCGAAGAATCA	Bely and Wray (2004)
*COII*	tRNA‐Asn‐CO2‐tRNA‐Asp: LumbF1	GGCACCTATTTGTTAATTAGG	Pérez‐Losada et al. (2009)
	tRNA‐Asn‐CO2‐tRNA‐Asp: LumbR2	GTGAGGCATAGAAATACACC	Pérez‐Losada et al. (2009)
*ND1*	tRNA‐Leu‐ND1‐LumbF2	GAATAGTGCCACAGGTTTAAAC	Pérez‐Losada et al. (2009)
	tRNA‐Leu‐ND1‐LumbR1b	TTAACGTCATCAGAGTTATC	Pérez‐Losada et al. (2009)
*12S rRNA*	12S‐tRNA‐Val‐16S‐LumbF1	CTTAAAGATTTTGGCGGTGTC	Pérez‐Losada et al. (2009)
	12S‐tRNA‐Val‐16S‐LumbR1	CCTTTGCACGGTTAGGATAC	Pérez‐Losada et al. (2009)
*16S rRNA*	16Sar	CGCCTGTTTATCAAAAACAT	Hillis (1996)
	16Sbr	CCGGTCTGAACTCAGATCACGT	Hillis (1996)

### 2.3 Data analysis

After assembly, all the resulting sequences of each gene were aligned using MAFFT programs with default parameters and manually edited to equal lengths (617 bp for *COI*, 725 bp for *COII*, 1000 bp for *12S rRNA*, 503 bp for *16S rRNA*, and 886 bp for *NDI*) using Geneious Prime v.2020.2.3 (http://www.geneious.com/). Gaps within the aligned sequences were treated as missing data. The sequences were checked against the GenBank database (http://www.ncbi.nlm.nih.gov/) using the BLASTn search algorithm to confirm species identity. In the analyses, genes or the concatenated sequences (*COI‐COII‐12S rRNA‐16S rRNA‐NDI*, 5 genes) were saved as different file types for various analysis programs.

The main genetic diversity indices, Tajima's *D* (Tajima, 1989) and Fu's *F*s (Fu, 1997) neutrality tests and mismatch distribution were calculated using DnaSP v.6 software (Rozas et al., 2017). *COI* gene, as the most common DNA barcoding for animal species identification, has been widely used not only to solve the evolutionary relationship among the closely related earthworm species but also to study the origin, differentiation and diffusion of various taxonomic categories of earthworm. In this study, to facilitate comparison with other studies, *COI* was used to calculate the degree of differentiation within the species. The extent of genetic differentiation among *A. aspergillum* from the different clades was assessed using two statistics. The first was the pairwise fixation index (Φ_ST_) estimated using Arlequin v.3.5 (Excoffier & Lischer, 2010), with significance determined by 10,000 data permutations. The second statistic was mean genetic distances based on the Kimura two‐parameter (K2P) model (Kimura, 1980) calculated in MEGA v.7 (Kumar et al., 2016) with 1000 bootstrap replicates. Based on the concatenated sequences haplotypes, the population structure was examined by a hierarchical analysis of molecular variance (AMOVA) with 10,000 permutations for statistical confidence implemented in the Arlequin v.3.5 program. Relationships among haplotypes (haplotype network) defined by concatenated sequences were constructed and visualized using the TCS method in PopART v.1.7 (Leigh & Bryant, 2015).

Phylogenetic analyses of the combined dataset of five mitochondrial genes, including Bayesian inference (BI) and maximum likelihood (ML), were conducted with *Metaphire daliensis* Yuan & Dong, 2019 as an outgroup (Yuan et al., 2019). The best‐fit model of evolution (TPM3uf + I + G) for concatenated sequences was estimated using Model Test v.2 (Posada, 2008) under the Akaike information criterion. ML analyses with bootstraps of 1000 replicates were run in PhyML v.3.0 (Guindon & Gascuel, 2003), and a BI tree was constructed using MrBayes v.3.2.6 in CIPRES (https://www.phylo.org). The relevant parameters were set as follows: the Markov chain was run for 20 million generations, sampling was performed once every 1000 generations, the first 10% of the obtained trees were removed as burn‐in, and random starting trees were used.

Divergence time estimation for *A. aspergillum* was performed using BEAST v.1.7 (Drummond et al., 2012) based on the uncorrelated relaxed clock and Bayesian statistics. Mutation rates of 2.1% per million years for *COI*, *COII*, and *ND1* genes and 1.2% for 12S and 16S genes were set (Chang et al., 2008; Chang & James, 2011; Novo et al., 2014; Pérez‐Losada et al., 2011). The root height of the tree between *M. daliensis* and *A. aspergillum* was set at 24.12 Mya (HPD: 20.82–27.90 Mya) owing to the lack of fossil records (Jiang & Qiu, 2018). Markov chains were run for 100 million generations, saving trees every 10,000th generation after a discarded burn‐in of 10,000,000 steps. Ancestral area reconstructions were performed using RASP v.3.2 (Yu et al., 2015). The samples were divided into eight regions (A–H) (Figure 2). The BI was performed using the Bayesian binary M chain Monte Carlo (BBM) model, and the maximum number of areas was set to two.

## 3 RESULTS

### 3.1 Mitochondrial sequence variation

Five mitochondrial DNA fragments (617 bp for *COI*, 725 bp for *COII*, 1000 bp for *12S rRNA*, 503 bp for *16S rRNA*, and 886 bp for *NDI*) were successfully amplified and sequenced from 157 *A. aspergillum* specimens collected at 75 locations in southern China. The final alignment of the combined datasets of five mitochondrial genes (in order: *COI*‐*COII*‐*12S rRNA*‐*16S rRNA*‐*NDI*) for all collected samples resulted in 3731 bp long sequences. The A + T base content (59.3%–68.1%) was higher than that of G + C (31.9%–40.7%) of each mitochondrial gene and the combined sequence, indicating an A + T bias consistent with that found in invertebrate mitochondria.

### 3.2 Population genetic structure of *A. aspergillum*

**FIGURE 3:**
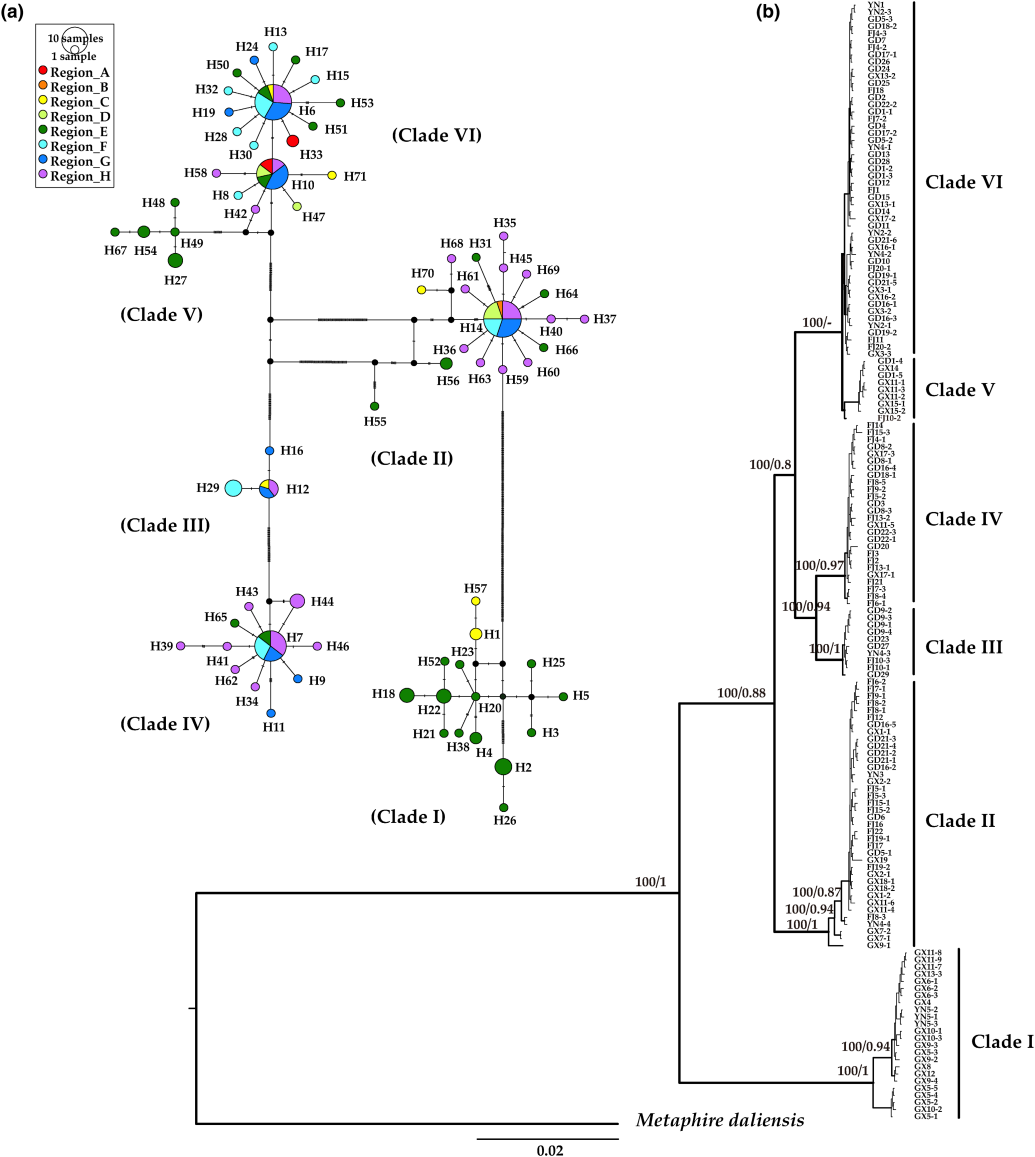
Haplotype network and phylogenetic tree of 157 individuals of *Amynthas aspergillum*. (a) TCS networks of mitochondrial concatenated sequences haplotypes. Each circle of network represents one haplotype, with size being proportional to the frequency. Black solid circles represent intermediate haplotypes that were not sampled or are extinct. (b) Bayesian inference tree based on concatenated sequences of five mitochondrial genes (*COI‐COII‐12S rRNA‐16S rRNA‐ND1*). Bayesian posterior probability (if ≥95%) and ML bootstrap values (if ≥80%) are shown above the branches.

Based on the combined mitochondrial sequences, the intraspecific phylogenetic relationships of *A. aspergillum* were constructed using BI and ML, with *M. daliensis* as the outgroup (Figure 3b). Six divergent clades (I–VI) were recovered using BI and ML with high posterior probability and bootstrap values, respectively. However, individuals within the clades were closely related, resulting in different intra‐clade topologies generated in the two analyses, both with weak support. There was no distinct phylogeographic structure among the clades of *A. aspergillum*. Clades II, IV, and VI showed a mixed geographical distribution, colonizing multiple provinces and regions (clades II and VI in four provinces and seven regions and clade IV in three provinces and four regions). Clades I and V were mainly distributed in the basin of south‐central Guangxi (region E). Clade III, with a small number of samples (10 individuals), was scattered in Yunnan (region C), Guangdong (regions F and G) and Fujian (region H). In many cases (18 of 38 populations with multiple samples), individuals from a single population were distributed in different clades, indicating large genetic differentiation within populations.

Among the 157 concatenated sequences with a length of 3731 bp, 71 haplotypes (H1–H71) were identified. The resulting TCS haplotype network showed six main groups of haplotypes corresponding to phylogenetic clades I–VI. The three larger groups (clades II, IV, and VI) consisted of three star‐like substructures dominated by haplotypes 14, 7, 6, and 10 (Figure 3a), indicating a population expansion. In addition, haplotypes 14 (*n* = 20), 7 (*n* = 14), 6 (*n* = 19), and 10 (*n* = 14) were shared by several individuals from multiple geographical regions, and they might be the ancestral haplotypes of their respective clades (Table 3).

**TABLE 3:** The distribution of haplotypes for *Amynthas aspergillum* based on combined datasets.

Region	Number of samples	Haplotype (number of individuals)
Region A	4	H10(2), H33(2)
Region B	1	H14(1)
Region C	7	H1(2), H6(1), H12(1), H57(1), H70(1), H71(1)
Region D	7	H10(2), H14(4), H47(1)
Region E	46	H2(4), H3(1), H4(2), H5(1), H6(2), H7(2), H10(2), H17(1), H18(3), H20(1), H21(1), H22(3), H23(1), H25(1), H26(1), H27(3), H31(1), H38(1), H48(1), H49(1), H50(1), H51(1), H52(1), H53(1) H54(2), H55(1), H56(2), H64(1), H65(1), H66(1), H67(1)
Region F	23	H6(5), H7(4), H8(1), H13(1), H14(4?), H15(1), H28(1), H29(4), H30(1), H32(1)
Region G	28	H6(6), H7(3), H9(1), H10(6), H11(1), H12(2), H14(6), H16(1), H19(1), H24(1)
Region H	41	H6(5), H7(5), H10(2), H12(2), H14(5), H34(1), H35(1), H36(1), H37(1), H39(1), H40(1), H41(1), H42(1), H43(1), H44(3), H45(1), H46(1), H58(1), H59(1), H60(1), H61(1), H62(1), H63(1), H68(1), H69(1)

### 3.3 Genetic diversity and differentiation of *A. aspergillum*

In this study, haplotype diversity (Hd) and nucleotide diversity (*π*) were mainly used to assess the level of genetic diversity in *A. aspergillum*. In general, *A. aspergillum* exhibited a high Hd (0.952 ± 0.008) and *π* (0.02691 ± 0.00178) (Table 4). Interestingly, a high Hd and low *π* were observed in the clades of *A. aspergillum*. Among them, clade I had the highest Hd (0.957 ± 0.025) and *π* (0.00310 ± 0.00060), and clade III had the lowest Hd (0.733 ± 0.101) and *π* (0.00025 ± 0.00006). AMOVA estimated the variance component of Hd at different levels of hierarchical division. The results (Table 5) revealed that 46.31% of the genetic variation occurred within populations (Φ_ST_ = 0.53688, *p* = .000), whereas 34.55% of the genetic variation occurred among populations within regions (Φ_SC_ = 0.42731, *p* = .000), and only 19.13% of the genetic variation occurred among regions (Φ_CT_ = 0.19133, *p* = .002). These findings indicate that molecular variance in *A. aspergillum* predominantly occurs at the population level.

**TABLE 4:** Genetic haplotype and nucleotide diversities of *Amynthas aspergillum* based on the concatenated sequences (*COI‐COII‐12S rRNA‐16S rRNA‐ND1*).

	Ns	Ls	Eta	S	Nh	Hd	*π*	Tajima's *D*	Fu's *F*s
Total	157	3731	376	357	71	0.952 ± 0.008	0.02691 ± 0.00178	1.294	9.430**
Clade I	24	3726	43	43	16	0.957 ± 0.025	0.00310 ± 0.00060	−0.147	−1.779
Clade II	38	3728	47	47	20	0.781 ± 0.073	0.00115 ± 0.00030	−2.245**	−8.837**
Clade III	10	3726	3	3	4	0.733 ± 0.101	0.00025 ± 0.00006	−0.431	−1.020
Clade IV	26	3726	18	18	13	0.757 ± 0.091	0.00044 ± 0.00011	−2.350**	−9.504**
Clade V	9	3726	13	13	6	0.889 ± 0.091	0.00095 ± 0.00039	−1.335	−0.840
Clade VI	50	3726	23	23	19	0.800 ± 0.043	0.00051 ± 0.00005	−2.093*	−14.324**

Abbreviations: Eta, total number of mutations; Hd, haplotype diversity; Ls, length of sequence; Nh, number of haplotypes; Ns, number of sequences; S, number of segregating sites; *π*, nucleotide diversity.

*p < .05; **p < .01.

**TABLE 5:** AMOVA results of *Amynthas aspergillum* populations among the regions using concatenated sequences (*COI‐COII‐12S rRNA‐16S rRNA‐ND1*).

Source of variation	df	Sum of squares	Variance components	Percentage of variation	*p*‐Value
Among regions	7	1626.253	9.34075	19.13	Φ_CT_ = 0.19133** (*p* = .002)
Among populations within regions	67	3798.016	16.86952	34.55	Φ_SC_ = 0.42731*** (*p* = .000)
Within populations	82	1853.967	22.60935	46.31	Φ_ST_ = 0.53688*** (*p* = .000)
Total	156	7278.236	48.81962		

**p < .01; ***p < .001.

According to the results of the phylogenetic and haplotype network analyses, genetic differentiation within and between the six clades of *A. aspergillum* was further evaluated based on the mitochondrial *COI* gene (Table 6). The K2P genetic distances ranged from 0.0020 (Clades V and VI) to 0.1026 (Clades I and IV) between clades, and 0.0001 (Clade IV) to 0.0076 (Clade I) within clades. Clade I had the greatest inter‐ and intra‐clade K2P genetic distances. Additionally, pairwise Φ_ST_ values among the six clades (0.8090–0.9903) were high and the differences were statistically significant.

**TABLE 6:** Measures of clade differentiation for *Amynthas aspergillum* obtained from *COI* data. Pairwise Φ_ST_ estimates (below diagonal) and mean genetic distances corrected with the Kimura‐2 parameter model (above diagonal) among the clades of *A. aspergillum*. Values in the diagonal with a bold font are average K2P distances within the clades.

	Clade I	Clade II	Clade III	Clade IV	Clade V	Clade VI
Clade I	**0.0076**	0.0994	0.1025	0.1026	0.0943	0.0966
Clade II	0.9654**	**0.0004**	0.0219	0.0218	0.0232	0.0253
Clade III	0.9435**	0.9839**	**0.0003**	0.0051	0.0182	0.0186
Clade IV	0.9610**	0.9857**	0.9815**	**0.0001**	0.0181	0.0168
Clade V	0.9362**	0.9817**	0.9903**	0.9897**	**0.0004**	0.0020
Clade VI	0.9697**	0.9836**	0.9820**	0.9820**	0.8090**	**0.0004**

Values in the diagonal with a bold font are average K2P distances within the clades.

**p < .01.

### 3.4 Divergence time and colonization history of *A. aspergillum*

**FIGURE 4:**
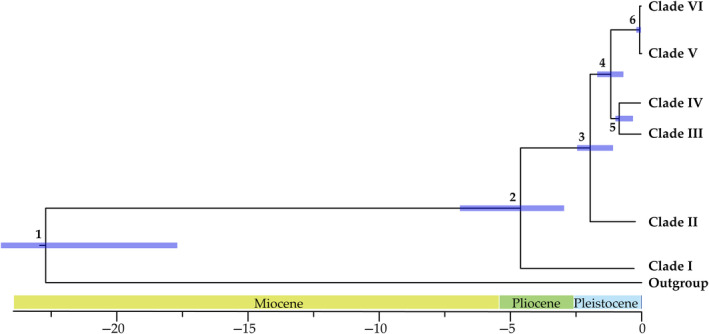
Divergence time estimate of *Amynthas aspergillum* based on concatenated sequences (*COI‐COII‐12S rRNA‐16S rRNA‐ND1*). Details of numbers in each node are shown in Table 7.

According to the divergence time analysis (Figure 4 and Table 7), the split between *A. aspergillum* and related species *M. daliensis* dated to 22.71 Mya (HPD: 17.70–24.41 Mya), consistent with previous findings (24.12 Mya, HPD: 20.82–27.90 Mya) (Jiang & Qiu, 2018). The primary diversification within *A. aspergillum* was estimated at 4.61 Mya (95% HPD: 2.95–6.92 Mya), when clade I split from the rest. Differentiation events of clades II–VI occurred during the Pleistocene (1.96–0.08 Mya). Reconstruction of the ancestral area showed that the basin of south‐central Guangxi (region E) may have been a key area for the origin and early major differentiation (clades I and II) of *A. aspergillum* in China. Over time, some *A. aspergillum* gradually dispersed toward the east and diversification events of clades III and IV, and clades V and VI occurred in regions G and H, respectively (Table 7).

**TABLE 7:** Ancestral areas reconstruction for *Amynthas aspergillum* recovered by BBM.

Node	BP	PP	Area	Pro	Divergence time (Mya)		
					Time	95% L‐HPD	95% U‐HPD
1	100	1.0	—	—	22.71	17.70	24.41
2	100	1.0	E	97.60	4.61	2.95	6.92
3	100	0.88	E	92.30	1.96	1.09	2.46
4	100	0.8	E	47.24	1.17	0.69	1.69
5	100	0.94	G	43.67	0.86	0.33	1.00
6	100	1.0	H	34.59	0.08	0.07	0.25

**FIGURE 5:**
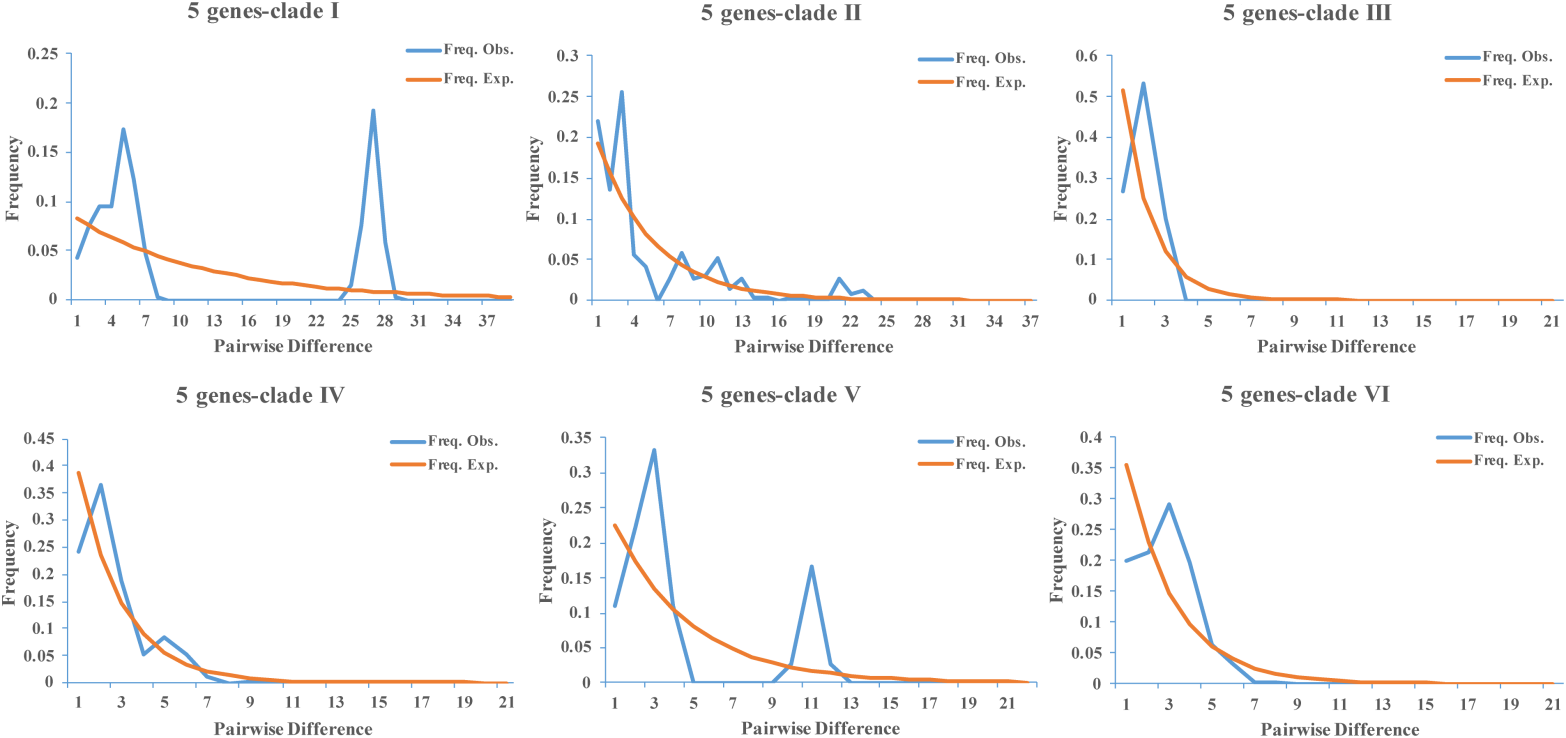
Mismatch distributions for *Amynthas aspergillum* and clades base on concatenated sequences (*COI‐COII‐12S rRNA‐16S rRNA‐ND1*). The abscissa represents the number of pairwise differences and ordinate represents frequency of pairwise comparisons. The blue line denotes observed frequencies and the orange line denotes the frequency expected under constant population model hypothesis.

Neutrality tests and mismatch distribution analyses were performed to deduce the demographic history. The neutrality tests revealed a few signs of demographic events in clades I, III, and V, whereas clades II, IV, and VI showed significant negative values in both tests, deviating from neutral mutations and possibly experiencing recent population expansion (Table 4). The mismatch distribution analysis of clades II, III, IV, and VI showed an approximately unimodal distribution, indicating a recent population expansion. Clades I and V showed a bimodal distribution, suggesting that the population size had been reduced or remained relatively stable (Figure 5).

## 4 DISCUSSION

The present study is the first to describe the genetic diversity and population structure of the medicinal earthworm *A. aspergillum*, which is widely distributed in southern China. Based on the mitochondrial genes, *A. aspergillum* showed a significantly high genetic variability. A total of 71 haplotypes were found, including five shared haplotypes and 66 unique haplotypes. Hd and *π* are commonly used to evaluate genetic diversity (Bonin et al., 2007). The overall Hd of *A. aspergillum* was 0.952 and *π* was 0.02691. A high genetic diversity is associated with a higher potential for natural selection, better environmental adaptability, and higher survival in the face of environmental change (Islam et al., 2019; Jin et al., 2022; Kangayan et al., 2022). The distribution area of *A. aspergillum* in China is mainly a hilly area south of the Yunnan‐Guizhou Plateau‐Nanling‐Wuyi Mountain Range. Thus, the better adaptation of *A. aspergillum* to habitat heterogeneity may be the reason for its higher genetic diversity.

High Hd and *π* levels may result from the presence of multiple differentiated lineages in a species, the contact of several independent populations, or a large and stable population that has evolved over a long period (Buckley et al., 2011; Fernández et al., 2016; Grant & Bowen, 1998). Our results support the alternative hypothesis of comparative differentiation. Six mitochondrial clades of *A. aspergillum* were detected by phylogenetic tree and haplotypes network analyses, and the strong genetic divergence was confirmed by genetic distance and Φ_ST_ values based on *COI* genes. The genetic distance of *COI* is generally used to measure the degree of genetic differentiation between and within taxonomic categories. In an evaluation of the genetic differentiation of 583 samples from the family Megascolecidae, Jiang reported genetic distance of *COI* ranged from 14% to 24% at the species level, 5% to 11% at the subspecies level, and 0% to 3% at the population level (Jiang & Qiu, 2018). Sun et al. (2017) found an average interspecies genetic divergence of 16.6% for *COI* in the genus *Amynthas*. The intraspecific *COI* genetic divergence ranges between 0.2% and 10% in *A. yuhsii* (Tsai, 1964) and between 2.9% and 10.3% in *A. formosae* (Michaelsen, 1922) (Chang & Chen, 2005). Dong et al. (2020) discovered that *COI* divergence between the two lineages of *A. triastriatus* (Chen, 1946) was 6.3%, together with certain morphological differences, suggesting the existence of a subspecies. In this study, *COI* genetic divergence among clades of *A. aspergillum* ranged from 0.2% to 10.26%. In particular, clade I had the greatest inter‐clade (9.43%–10.26%) and intra‐clade genetic divergence (0.76%), suggesting the existence of cryptic species. However, population size, rate of mutation and divergence time vary between species, a universal fixed distance threshold may not be applicable to all species (Lin et al., 2021; Yang & Rannala, 2017). Another more widely‐used and rigorous threshold for species delimitation is the “10× rule”—the mean interspecific distance is considered to be at least 10 times as large as the mean intraspecific distance (Hebert et al., 2004). We applied the “10× rule” to identify situations where clade I might represent a cryptic species. Based on the cryptic species hypothesis in this study, the average intraspecific distance was 1.42%, and the average distance between clade I and clades (II–VI) were 9.88%, which was less than 14.2% (1.42% × 10). Therefore, it failed to discriminate clade I as a cryptic species according to “10× rule”. Both methods try to find a “barcoding gap”, a significant difference between intraspecific variation and interspecific variation in *COI* sequences (Hebert et al., 2004). However, with the expansion of geographical range and the addition of a larger proportion of closely related species, the increase of intraspecific distance and the decrease of interspecific distance are likely to reduce the efficiency of DNA barcoding delimitation (Cai et al., 2010). This makes the existence of barcoding gap controversial. Critics even argued that the barcode gap is a temporary result of insufficient sampling between taxa and individuals (Meyer & Paulay, 2005). Conversely, it has also been shown that extensive geographic sampling, while altering the values of intra‐ and inter‐species nucleotide divergence, does not affect the success of barcoding (Gaikwad et al., 2012; Lukhtanov et al., 2009). In fact, this helps to identify cryptic species (Dong et al., 2020; Hebert et al., 2010; Jin et al., 2022). Despite these controversies, sequence divergence based on DNA barcoding remains the most universally effective molecular technique for species delimitation. But the term barcode gap requires a more precise and statistically sound definition (Čandek & Kuntner, 2015). For species with deep intraspecific divergence, inadequate sampling, incomplete lineage sorting and gene flow may lead to incorrect species delimitation (Lin et al., 2021). Therefore, the final species delimitation requires further comprehensive validation from both morphological and molecular aspects, or even in combination of other types of information such as geography, behavior, and ecological preferences (Cheng et al., 2023).

According to the estimation of divergence time and the reconstruction of ancestral areas based on mitochondrial genes, the results indicated that *A. aspergillum* originated from southern Guangxi in the early Miocene and differentiated into clades I and II in the early Pliocene and early Pleistocene, respectively. The split between clade III and IV dated to approximately 0.86 Mya in region G. Clades V and VI diversion dated to approximately 0.08 Mya in region H. The ancestral area of the clades ranged from E to G and finally to H, indicating that *A. aspergillum* gradually dispersed toward the east after its origin in Guangxi, and that the Yunnan‐Guizhou Plateau, Nanling, and Wuyi Mountains might be the geographical obstacles for its spread to the west and north. Interestingly, the current distribution areas of clades II, VI, and VI were much larger than their ancestral distribution areas, indicating the occurrence of population expansion events (Siqueira et al., 2013). The conjecture of a recent demographic expansion event is supported by our results, including the significant negative values of neutrality tests and an approximately unimodal mismatch distribution (Pilkington et al., 2008). Additionally, the patterns of genetic diversity observed in clades of *A. aspergillum*, such as the high Hd and low *π* levels, combined with star‐shaped haplotype networks and a high number of less frequent haplotypes, are characteristic of clades that have undergone recent demographic expansion (Albernaz et al., 2012; Excoffier et al., 2008; Searle, 2000; Wares, 2010). This study is the first to explore the genetic diversity and genetic structure of *A. aspergillum*，and its preliminary findings are yet to be tested in future studies. We look forward to adding more geographic samples and nuclear gene or genomic data based on this study to provide new insights into the differentiation and dispersal of *A. aspergillum*.

## 5 CONCLUSIONS

*Amynthas aspergillum* (Perrier, 1872) is a natural resource that is widely distributed in southern China, which is used in traditional Chinese medicine (Guang‐dilong). This study is the first report on the basic genetic diversity and structure information of *A. aspergillum* using the mitochondrial DNA analyses. The results indicated a high degree of genetic variation within and between populations of *A. aspergillum*, identifying six mitochondrial clades (Clades I–VI). Notably, individuals from clades II, IV, and VI were widely distributed geographically, covering the entire distribution area of this species. This may be because these three clades have recently undergone demographic expansion. Additionally, divergence time estimates and the reconstruction of the ancestral area indicated that *A. aspergillum* originated in Guangxi. Subsequently, it underwent initial intraspecific diversification in the early Pliocene, formed clade I, gradually dispersed eastward in the Pleistocene, and rapidly differentiated into clades II–V. The Yunnan‐Guizhou Plateau, Nanling Mountain, and Wuyi Mountain may act as geographical barriers to the westward and northward spread of *A. aspergillum*. In the future, we aim to add more samples, incorporate nuclear gene information, and further explore the existence of cryptic species and the causes of the existing geographical lineage distribution pattern of this species based on this study.

## AUTHOR CONTRIBUTIONS

**Jiali Li:** Conceptualization (equal); data curation (equal); formal analysis (equal); investigation (equal); methodology (equal); validation (lead); visualization (lead); writing – original draft (equal); writing – review and editing (equal). **Jibao Jiang:** Conceptualization (equal); data curation (equal); formal analysis (equal); funding acquisition (equal); investigation (equal); methodology (equal); project administration (equal); supervision (equal); writing – original draft (equal); writing – review and editing (equal). **Qing Jin:** Data curation (equal); investigation (equal). **Zhu Yuan:** Conceptualization (equal); funding acquisition (equal); investigation (equal); methodology (equal); project administration (equal). **Jiangping Qiu:** Conceptualization (equal); funding acquisition (equal); methodology (equal); project administration (equal); supervision (equal); writing – original draft (equal); writing – review and editing (equal).

## FUNDING INFORMATION

This study was supported by National Nature Science Foundation of China Grant No. 42077028, No. 42101066, No. 41701272, No. 41471204, and National Science & Technology Fundamental Resources Investigation Program of China No. 2018FY100300.

## CONFLICT OF INTEREST STATEMENT

The authors have no conflicts of interest to declare.

## Data Availability

The mitochondrial DNA of *A. aspergillum* used to support the findings of this study were deposited in the GenBank database for the purpose of making the data widely available to the scientific community.
